# Iterative Navigator-Based Retrospective Motion Correction for Multi-Shot Brain MR Elastography and Evaluation of Single-Shot Post-Reconstruction Correction

**DOI:** 10.1002/mrm.70625

**Published:** 2026-10-09

**Authors:** Zhuoyu Shi, Alex Michael Cerjanic, Grace McIlvain

**Affiliations:** 1 Department of Biomedical Engineering, Fu Foundation School of Engineering and Applied Science, Columbia University, New York, New York, USA; 2 Department of Neurology, Massachusetts General Hospital, Boston, Massachusetts, USA; 3 Department of Radiology, Vagelos College of Physicians and Surgeons, Columbia University, New York, New York, USA

**Keywords:** brain magnetic resonance elastography, brain tumor, image reconstruction, mechanical property, motion correction

## Abstract

**Purpose:**

Brain MR elastography (MRE) is uniquely sensitive to subject motion, as even small phase deviations can produce large errors in the mechanical properties estimated. Existing motion correction strategies have not been validated for MRE and are often incompatible with complex multi-shot or joint temporal reconstructions.

**Methods:**

We propose COMET (Correction Of Motion in Elastography via Transformation), a framework that estimates rigid-body motion from navigator images and embeds transformation matrices into an iterative reconstruction and displacement-vector reorientation for multi-shot MRE. We additionally validate post-reconstruction correction and displacement-vector reorientation for single-shot MRE. The framework was evaluated on MRE from 9 pairs of EPI single-shot and 9 pairs of spiral multi-shot acquisitions, each containing a with-motion scan and a reference. Additionally, four representative motion patterns with increasing amplitude were simulated to indicate how different motions affect MRE quality and results, and a brain tumor MRE dataset with simulated motion was used to assess clinical effect.

**Results:**

Motion correction improved stiffness accuracy across all tested datasets, reducing average stiffness errors by up to 240.86% in multi-shot spiral MRE, and by up to 44.28% in single-shot EPI, across in vivo motion ranging from 4.81 to 67.58 mm translation and 1.85° – 23.63° rotation. In a pediatric glioma dataset, simulated motion completely obscured tumor stiffness contrast, while correction restored tumor stiffness visibility. Robustness of estimated stiffness maps depended on motion amplitude, temporal motion patterns, and encoding strategies.

**Conclusion:**

This approach enables robust motion correction for single- and multi-shot brain MRE, and expands reliable MRE to clinical and challenging populations.

## 1 | Introduction

Magnetic resonance elastography (MRE) is an MRI technique that noninvasively measures the mechanical properties of soft tissue in vivo, thereby assessing the integrity of microstructural architecture. This technique has been applied to a range of neurological conditions, including brain tumors [1], aging [2], and multiple sclerosis [3]. MRE encodes micron-level tissue displacement from harmonic mechanical vibrations into the phase component of the MR image and generates quantitative stiffness maps by finding the inverse solution to the viscoelastic wave equation [4]. Therefore, the accuracy of mechanical property inversions relies on the fidelity of the phase image.

A major challenge across all MR imaging is the impact of subject motion on image quality. MRE is uniquely sensitive to subject motion, as even small head movements can introduce phase inconsistencies that degrade the measured displacement fields and propagate as bias into the mechanical property maps. Even when the magnitude image appears visually accurate, the quantitative MRE mechanical property maps may contain errors that affect interpretation. Additionally, MRE requires a long acquisition time to collect multiple motion-encoding directions and temporal offsets, making it inherently long, and posing particular difficulties when scanning challenging populations, such as children [5].

Accurate motion estimation is essential for effective correction and can be enabled by either auxiliary hardware or sequence-embedded strategies. External approaches, such as optical tracking systems [6, 7], have been designed to assist real-time motion estimation within the scanner. These approaches are shown to estimate motion accurately and improve image quality through corresponding motion compensation approaches [6, 7]. However, external techniques are limited in their generalizability across scanner types and sites due to reliance on additional hardware that must be designed to fit within the scanner and avoid introducing artifacts or interfering with radiofrequency fields [6, 7]. Therefore, sequence-embedded motion estimation approaches have been developed. These include self-navigated methods, which estimate motion parameters by aligning images or *k*-space data [8], and navigator-based methods, which acquire low spatial resolution at the same temporal resolution as images to track motion [9–12].

With accurate motion parameter estimates, correction can be tackled via prospective or retrospective approaches. Prospective strategies update imaging gradients, RF pulses, and slice positions in real time to maintain alignment between the imaging coordinates and the moving anatomy [6, 12]. While these approaches preserve *k*-space consistency and prevent motion artifacts before they become embedded into the image, they are often difficult to generalize across scanner vendors due to their reliance on vendor-specific pulse-sequence implementations and hardware integration [13]. Retrospective correction, on the other hand, compensates for motion after image acquisition, including post-reconstruction correction, *k*-space correction [14], and iterative motion-aware reconstruction [10, 15].

Despite substantial progress in MRI motion correction strategies, approaches tailored to MRE remain limited. Recent work introduced a retrospective motion and susceptibility distortion correction framework for EPI-based brain MRE [16], and showed reduced point-spread blurring and improved gray-white matter delineation. However, this approach was limited to single-shot EPI acquisitions and did not account for motion-induced changes in displacement vectors. Emerging MRE methods leverage multi-shot imaging [17] and joint-timepoint reconstruction sequences [18] to improve spatial resolution, SNR, and scan efficiency. These techniques combine data from multiple shots or time points via iterative reconstruction, in which any misalignment between shots propagates throughout the reconstruction, leading to global image degradation [19]. Consequently, motion correction for multi-shot phase-based imaging must be incorporated within the reconstruction framework itself, rather than being applied after image reconstruction.

Here, we propose COMET (Correction Of Motion in Elastography via Transformation), a framework that retrospectively integrates motion-compensation into iterative reconstruction and displacement-vector reorientation for multi-shot and joint-reconstruction acquisitions, and we demonstrate the efficacy of post-reconstruction correction and displacement-vector reorientation for single-shot MRE. We also simulated human motion to understand how motion patterns and amplitudes affect the amount of error in mechanical property maps. By adapting motion estimation and correction techniques specific to multi-shot or single-shot MRE, the proposed framework reduces motion artifacts, enhances image quality, and improves the robustness of mechanical measures across all types of MRE data acquisition.

## 2 | Theory

The proposed method estimates and incorporates rigid-body motion directly into the iterative reconstruction model, enabling motion-compensated image reconstruction. Navigator images associated with each shot and slice are registered to a reference navigator to estimate the rigid-body transformation matrix T, which is then scaled to account for the differing resolution between the low-resolution navigators and high-resolution MRE images. Since rigid-body transformations preserve distances and have a non-zero determinant, T is inherently invertible and $T^{-1}$ is well-defined. The inverse transformation was applied to the real and imaginary image components using interpolation during forward model evaluation. For shot t, the measured k-space data are modeled as:

$$y_{t}=A_{t}SP_{t}T_{t}^{-1}x+\eta_{t},$$

where x is the unknown complex image, $T_{t}^{-1}$ is the inverse scaled rigid-body transformation matrix for shot $t,P_{t}$ is the shot-dependent phase-error correction implemented as complex phase modulation derived from the corresponding navigator phase, S denotes coil sensitivity maps [4], $A_{t}$ is the Fourier encoding system matrix including field inhomogeneity, implemented using a Non-Uniform Fast Fourier Transform (NUFFT) with the time segmentation algorithm [20], and $\eta_{t}$ is measurement noise that is assumed to follow a zero-mean complex Gaussian distribution [20].

To maintain mathematical consistency and stable convergence, the adjoint model was defined as:

$$x_{\mathrm{adj}}={\left(T_{t}^{-1}\right)}^{H}P_{t}^{H}S^{H}A_{t}^{H}y_{t},$$

which back-projects the k-space data through the Fourier encoding matrix with field inhomogeneity correction, combines coil channels, removes the estimated phase error, and maps the data into the common reference space. Embedding the transformation matrices in both forward and adjoint operations ensures model consistency and convergence within iterative reconstruction [20].

The final motion-aware image reconstruction can thus be formulated as:

$$\overset{ˆ}{x}=arg\underset{x}{min}\frac{1}{2}\sum_{t=1}^{N}{\left\|A_{t}SP_{t}T_{t}^{-1}x-y_{t}\right\|}_{2}^{2}+\beta \|ℛx{\|}_{2}^{2},$$

where x is the unknown complex image, N is the total number of shots, and ℛ is a regularization operator enforcing quadratic spatial smoothness with a regularization factor β. The resulting penalized least-squares problem could not be solved explicitly in closed form due to the large-scale and computationally intensive operators, so it was solved using a preconditioned conjugate gradient (PCG) algorithm with 15 iterations.

To account for head-motion-induced changes in the effective motion-encoding directions, an additional displacement-vector reorientation step was incorporated into the post-processing pipeline for both single-shot and multi-shot MRE. For each motion-encoding direction d and temporal phase offset p, the positive and negative encoding polarity images were combined as a complex-valued ratio:

$$c_{d,p}(r)=\frac{{\overset{ˆ}{x}}_{d,p,+}(r)}{{\overset{ˆ}{x}}_{d,p,-}(r)},$$

where r denotes spatial position, ${\hat{x}}_{d,p,+}(r)$ and ${\hat{x}}_{d,p,-}(r)$ are the reconstructed motion-corrected complex images acquired with positive and negative motion-encoding polarities, respectively. The phase of $c_{d,p}(r)$ was extracted and unwrapped to obtain $z_{d,p}(r)$, which is the displacement-encoded phase measurement for encoding direction d and temporal phase offset p.

For each estimated transformation matrix, the rotational component was extracted using singular value decomposition. Because the motion-encoding gradients are fixed in scanner coordinates while the head rotates, the original motion encoding directions $e_{\mathrm{ori,d}}$ were then transformed into effective encoding directions $e_{\mathrm{eff,d,p}}$ by:

$$e_{eff,d,p}=R_{d,p}^{T}e_{ori,d},$$

where $R_{d,p}$ is the rotational component of the estimated head-motion transform at phase offset p and encoding direction *d*. For each phase offset p, the unwrapped phase measurements from the three motion-encoding directions were stacked as $z_{p}(r)$, the effective encoding directions were stacked into a matrix $E_{\mathrm{eff},p}$, and the reoriented displacement-encoded phase vector was calculated as:

$$u_{p}(r)=E_{eff,p}^{+}z_{p}(r),$$

where + denotes the Moore-Penrose pseudoinverse, $z_{p}(r)$ is a column vector containing the three unwrapped displacement-encoded phase measurements across three motion-encoding directions, and $u_{p}(r)$ is the reoriented displacement-encoded phase vector. $u_{p}(r)$ was then Fourier transformed across temporal phase offsets to estimate the complex first-harmonic displacement field:

$$U(r)={ℱ}_{p}{\left\{u_{p}(r)\right\}}_{1}.$$

This correction compensates for head-motion-induced changes in the encoding geometry and reconstructs the displacement-encoded data in a consistent anatomical coordinate system before mechanical inversion.

## 3 | Methods

### 3.1 | Image Acquisition

This study was approved by the Columbia University Institutional Review Board, and all participants gave informed written consent. Six healthy young adult participants (four females and two males; age 21–30 years) were scanned multiple times on a Siemens 3T Prisma scanner (Siemens Medical Solutions; Erlangen, Germany) using a 64-channel RF-receive head coil. Image acquisition included single-shot EPI and multi-shot spiral MRE. For all MRE scans, vibrations were delivered to the posterior of the skull at 50 Hz using a pneumatic actuator system (Resoundant Inc.; Rochester, MN) with a passive pillow driver using 15% amplitude. Bilateral, flow-compensated bipolar motion encoding gradients at 70 mT/m were synchronized to the period of vibration to encode displacement into the phase signal of the MRI. For both MRE sequence types, three directions of motion encoding were used.

The EPI MRE scans were acquired using a single-shot 2D spin-echo sequence [21] at 2.5 × 2.5 × 2.5 mm^3^ resolution with four or eight evenly spaced phase offsets. Additional imaging parameters include: 240 × 240 mm^2^ field of view (FOV); matrix size 96 × 96, 48 slices, TR/TE = 6720/69 ms, GRAPPA *R* = 3. The acquisition time was 3 min 15 s for a single-shot four-offset EPI MRE scan and 6 min 30s for a single-shot eight-offset EPI scan.

The spiral MRE scans were acquired using a 3D multiband, multishot spiral spin-echo sequence [22] with $2.0\times 2.0\times 2.0{\;mm}^{3}$ resolution. The k-space sampling trajectories were designed to have $4k_{z}$-planes and 4 in-plane $k_{xy}$-shots. Data was collected with SENSE parallel imaging [23] undersampling both in-plane and through-plane ($R_{xy}=2$ and $R_{z}=2$). Additional imaging parameters included: $240\times 240{\;mm}^{2}$ FOV; 120×120 matrix size; 64 slices; TR/TE=2240/70ms; and 4 evenly spaced phase offsets. The multiband MRE acquisition time was 3 min 35 s. Additionally, a $B_{0}$ field mapping scan was separately collected for each spiral scan with the same number of slices as image data, with parameters including: $240\times 240{\;mm}^{2}$ FOV; 120×120 matrix size; 64 slices; and $TR/{TE}_{1}/{TE}_{2}=3200/15.0/15.9\;ms$. The resulting field map was subsequently incorporated into the multi-shot spiral iterative reconstruction to correct for off-resonance effects. Low-resolution 3D navigator images (matrix 40×40×4) with the same FOV were acquired using a spiral-in trajectory blipped between $k_{z}$-planes, sampled once per spiral readout, and have the same phase, contrast, and object orientation as the MRE image data to which they belong [18, 22].

During each scanning session, a pair of EPI or spiral MRE scans was collected, including a no-motion reference scan and a with-motion scan. During motion scans, subjects were asked to move and stay in the new position every 40 s, including staying still for the first 40 s, shifting head to left/right and staying for the second 40 s, coming back for the next 40 s, rotating head to left/right and staying for 40 s, and coming back for the rest of the scanning time. Each of the six participants was scanned, with three of the participants being scanned on 2 days for a total of 9 pairs of with- and without-motion EPI scans and 9 pairs of with- and without-motion spiral scans. An additional EPI eight-phase offset scan and six additional spiral scans were acquired from one of the healthy female participants for *Experiments 1* and *2*, respectively.

### 3.2 | Image Processing

The EPI scans were reconstructed on the scanner. The spiral scans were reconstructed off-line using an iterative reconstruction based on a phase-corrected SENSE algorithm that includes correction for B_0_ field inhomogeneities [20] and motion-induced phase errors [22, 24]. The forward encoding operator consisted of the non-uniform Fourier encoding defined by the spiral sampling trajectory with B_0_ off-resonance correction implemented using the standard time-segmentation formulation [20]. Specifically, for each multishot spiral MRE acquisition, the separately acquired B_0_ field map, spiral k-space trajectory, and acquisition timing information were used to construct the time-segmented off-resonance encoding operator following Sutton et al. using four temporal segments [20], which are used to approximate off-resonance phase evolution during spiral readout. During reconstruction, this operator was applied in both the forward and adjoint operations to account for B_0_-induced phase evolution. Motion-induced phase errors were corrected using the phase-corrected SENSE framework [22, 24].

The with-motion EPI and spiral scans were reconstructed a second time using the corresponding motion-compensation techniques as described in the following sections. A pipeline figure with all intermediate steps from a single-shot EPI dataset and a multishot spiral dataset with real motion is included in the Supporting Information (Figure S1). Reconstruction was performed in MATLAB and required approximately 8h per dataset on a workstation equipped with a 24-core CPU. Incorporating motion correction into the reconstruction did not substantially increase the reconstruction time.

MRE displacement fields and subsequent mechanical property maps were determined from the reconstructed phase images. FMRIB Software Library (FSL) PRELUDE [25] was used to unwrap MRE phase images, and temporal Fourier filtering was used to determine complex harmonic displacement fields [26]. From the displacement fields, a nonlinear inversion algorithm (NLI) [4] was used to calculate mechanical property maps from the complex viscoelastic shear modulus G=G′+iG″, with G′ as the storage modulus and G″ as the loss modulus, and shear stiffness, μ, as $\mu =2|G{|}^{2}/\left(G\prime +|G\right)$ [26].

### 3.3 | Image Analysis

OSS-SNR was calculated for all reconstructed images to evaluate image quality, with OSS-SNR > 3.0 is considered sufficient strain quality for inversion [27]. Motion corruption is expected to decrease OSS-SNR, as motion-induced phase inconsistencies disrupt the displacement field and reduce the temporal and spatial coherence of the encoded wave signal, leading to lower effective signal-to-noise. Motion effects were quantified using the normalized root mean square error (NRMSE) and average stiffness error (AVSE) between no-motion and with-motion stiffness maps and between no-motion and motion-corrected stiffness maps, computed over the entire brain volume. Regional gray matter (GM) and white matter (WM) AVSE were calculated from FSL FAST [28] generated tissue masks thresholded at 0.6, using the no-motion stiffness map as the reference.

### 3.4 | Experiment 1: Examination of Motion Corruption on MRE Mechanical Property Maps

To systematically examine how different motion thresholds and patterns degrade MRE mechanical property maps, we simulated in-plane motion on the eight-offset EPI MRE data acquired on a healthy young-adult female. Because standard MRE scans often use either 8 or 4 phase offsets, we evaluated motion effects on both by analyzing the full eight-offset dataset and a subset containing four offsets (offsets 2, 4, 6, and 8).

Motion was simulated by translating (*x*-translation, *y*-translation) and rotating the image at corresponding MRE time points, under four representative motion patterns: drift, erratic, move-once-return, and move-once-stay. For a given simulation, the prescribed maximum amount of motion was 1.0, 2.5, 5.0, 7.5, and 10mm in the *x* or *y* direction and rotations of 2°, 5°, 10°, 15°, and 20°. The simulated patterns included: (i) drift movement, with each time point being shifted an incremental amount between zero and the maximum, (ii) erratic movement, with each time point being shifted a random amount between zero and the maximum, (iii) move once and return, with a single time point being shifted the maximum amount, (iv) move once and stay, with the first half of the time series moved zero and the second half moved the maximum.

To demonstrate the clinical effect, we simulated motion on a previously acquired and published pediatric brain tumor dataset from an 11-year-old male with a contrast-enhancing low-grade glioma [1]. A 10mm in-plane *x*-direction translation was applied to the second half of the timepoints to mimic abrupt mid-scan motion and stay, and post-reconstruction motion correction and reorientation were then applied.

### 3.5 | Experiment 2: Examination of Head-Position-Dependent Wave Pattern Variability

To evaluate whether head position relative to the passive driver alters stiffness estimates beyond rigid-body alignment effects alone, one healthy participant was scanned under small- and large-repositioning conditions in three controlled head positions: neutral, left-rotated, and right-rotated. No deliberate motion occurred during any individual acquisition; instead, the participant repositioned their head between scans and remained still at each prescribed position. Stiffness maps were reconstructed independently for each condition. AVSE between stiffness maps was then calculated to quantify wave-pattern variability.

### 3.6 | Experiment 3: Iterative Reconstruction Motion Correction

Nine pairs of multi-shot spiral with- and without-motion scans were reconstructed and corrected using the proposed iterative correction with and without displacement-vector reorientation, as described in Section 2. Navigator images from each shot were registered to the first navigator repetition as a reference using MATLAB (MathWorks, Natick, MA) cross-correlation to estimate rigid-body transformation matrices. The largest translation and rotation among the set were recorded as the maximum amount of subject motion, and the motion effects were analyzed by calculating AVSE, as described in Section 3.3.

### 3.7 | Experiment 4: Post-Reconstruction Motion Correction

Additionally, nine sets of single-shot EPI data were collected with a no-motion reference and a with-motion scan, using the same motion pattern as for the spiral datasets. For each of these datasets, post-reconstruction correction was applied with and without displacement-vector reorientation. The magnitude image of each repetition was rigidly registered to the first reference repetition using FSL FLIRT [29] with 6 degrees of freedom, and the resulting matrices were applied to the real and imaginary components of the corresponding complex images. The largest translation and rotation were extracted from the matrices for each scan to reflect the amount of motion from each subject, and the motion effect analysis described in Section 3.3 was implemented.

## 4 | Results

### 4.1 | Experiment 1: Examination of Motion Corruption on MRE Mechanical Property Maps

Table 1 demonstrates the severity of motion patterns and thresholds on the quality of mechanical property maps for the simulated motion corruption on EPI data. Across all simulations, erratic motion induced the largest errors in both the 8-offset image and the 4-offset subset. This instability was most evident at 20° rotation, which generated an NRMSE of 343.59% and an AVSE of 55.30%, indicating significant corruption of anatomical structures and wave displacements. In contrast, the move-once-return pattern was more resilient, producing errors below 10% even at moderate-to-large amplitudes. Drift motion and move-once-stay induced intermediate levels of error, corresponding to an NRMSE range of 2.43%–48.60% and AVSE range of 0.65%–17.79% in the 8-offset image and the 4-offset subset. In addition, comparing encoding strategies revealed additional differences. The 8-offset EPI dataset demonstrated greater resilience to transient motion (move-once-return and move-once-stay), while the 4-offset subset performed better under sustained motion (drift motion and erratic motion). Overall, all patterns showed an ascending trend in errors with increasing translations and rotations.

Figure S2 shows the representative clinical case, in which simulated motion blurred the lesion, eliminated stiffness contrast, and increased the average error by 39.91%. Lesion stiffness changed from 2.32 kPa without motion to 5.66 kPa with motion, and was returned to 2.32 kPa after correction. Visible tumor contrast was restored, and image quality was improved from OSS-SNR < 3 pre-correction to OSS-SNR > 3 after correction.

### 4.2 | Experiment 2: Examination of Head-Position-Dependent Wave Pattern Variability

Figure 1 demonstrates that changes in head position relative to the location of the passive driver resulted in AVSE ranging from 0.46% to 3.34% under small-reposition conditions (10.82–13.04 mm translation and 4.05°–4.30° rotation) and 2.81%–3.25% under large-reposition conditions (47.37–54.15 mm translation and 17.89°–18.78° rotation).

### 4.3 | Experiment 3: Iterative Reconstruction Motion Correction

In the multi-shot spiral acquisitions, detected motion ranged from 6.77 to 67.58 mm in translation and from 2.98° to 23.63° in rotation, resulting in stiffness errors of 3.14% to 269.69% (Figures 2 and 3). Uncorrected multi-shot images showed blurring brain regions (e.g., ventricles in Scan 1 magnitude image) and shot-to-shot intensity inconsistencies in magnitude images and widespread artifact propagation in stiffness maps (e.g., red spots in Scan 1 stiffness map). Some scans appear to have good magnitude images and OSS-SNR before correction, but produced stiffness maps with errors. For example, Scan 3 seems to have a good magnitude image before correction with OSS-SNR = 4.02, but there are red spots in its stiffness maps. Applying the proposed iterative correction framework significantly restored image sharpness and gray-white matter boundary, and increased OSS-SNR in nearly all cases. Stiffness map accuracy was improved in every test case, with an average increase of 0.37%–240.86% after correction, depending on the severity of the initial corruption. In a representative case (Scan 1) with mild-to-large motion (25.88 mm translation and 10.91° rotation), the image was recovered from 8.11% AVSE to just 0.04% AVSE, removing the propagated motion stiffness error and recovering a stiffness distribution very similar to the reference distribution. Scan 8 was the most corrupted dataset, with an AVSE of 269.69%, which was reduced to 28.83% after correction. The largest motion (67.58 mm and 23.63°) was detected in Scan 7, which was recovered from an unusable scan (OSS-SNR < 3) to usable quality (OSS-SNR > 3) after correction. Figure S3 illustrates the independent impact of displacement-vector reorientation from image alignment. Motion correction improved stiffness estimation in both GM and WM (Table S1), with comparable levels of improvement across tissue types. Overall, the proposed framework did not negatively affect the accuracy of the whole-brain stiffness maps in any of the observed spiral test cases.

### 4.4 | Experiment 4: Post-Reconstruction Motion Correction

As seen in Figures 4 and 5, in single-shot EPI scans, detected motion ranged 4.81–37.93 mm translations and 1.85°–14.14° rotations, introducing 1.80%–57.31% stiffness error, which post-reconstruction correction reduced to 1.07%–17.36%. Uncorrected single-shot images showed blurring brain regions in magnitude images (e.g., ventricles in Scan 4 magnitude image) and widespread artifact propagation in stiffness maps (e.g., red spots in Scan 4 stiffness map). Some scans appear to have good magnitude images and OSS-SNR before correction, but produced stiffness maps with errors. For example, Scan 9 has clear anatomical boundaries in the magnitude image before correction, with OSS-SNR = 3.77, but has red spots in the stiffness map. Applying the post-reconstruction correction framework significantly restored image sharpness and gray-white matter boundary, and increased OSS-SNR in nearly all cases. Stiffness map accuracy was increased by 0.44%–44.28% after correction, depending on the severity of the initial corruption. In a mild-to-large motion case (Scan 5) with 24.11 mm translation and 8.78° rotation, the motion-induced shift in ventricles was visible in the magnitude image, and this error propagated into significant stiffness errors shown as hot spots in the stiffness map. The corrected image showed a stiffness distribution very similar to the reference distribution. Scan 9 had the largest detected motion (37.93 mm and 13.92°), with an AVSE of 51.29% reduced to 17.36% after correction. Figure S4 illustrates the independent impact of displacement-vector reorientation from image alignment. Motion correction improved stiffness estimation in both GM and WM (Table S1), with comparable levels of improvement across tissue types. Notably, all scans with OSS-SNR < 3 (Scans 7 and 8) were recovered to OSS-SNR > 3 after correction, and the proposed framework did not have a negative effect on the accuracy of the whole-brain stiffness maps in any of the observed test cases.

## 5 | Discussion

Here we provide a systematic look at subject motion corruption and correction in brain MRE. We evaluate the degradation of mechanical property maps from subject motion and wave pattern changes, present a method for single-shot post-reconstruction correction and displacement-vector reorientation, and a framework for motion correction during iterative reconstruction that can bypass the challenges faced with motion correction of a multi-shot or temporally jointly-reconstructed sequence. We demonstrate the importance of assessing all MRE data for subject motion prior to publication, given that errors can occur in mechanical property estimations even when the magnitude image appears visually clear. We find that subject motion results in distinct mechanical-measure disruptions depending on the motion patterns and encoding strategies, and the post-reconstruction and iterative correction approaches improve the accuracy of property maps in all single-shot and multi-shot cases tested.

Subject motion is a widespread problem in MRI, particularly for challenging populations such as children and older adults with chronic pain. A previous study has reported that 35% of clinical pediatric brain MRI scans required repeat acquisition due to motion-induced image degradation [30]. As brain MRE becomes more widely used in these populations [1, 2], the understanding of the effects of subject motion on the accuracy of resulting property maps is essential. Since MRE relies on phase information and mechanical inversion, even small uncorrected motion can introduce systematic errors that meaningfully bias study outcomes. This challenge is amplified in brain MRE compared to conventional MRI because MRE relies on combining wave information acquired across multiple temporal offsets. Subject motion occurring at different temporal offsets can therefore introduce inconsistencies in the reconstructed wave field, which subsequently propagate into the mechanical inversion and degrade the resulting stiffness maps. Notably, in many mild-motion cases shown in our results, the brain magnitude images may appear acceptable, but the resulting stiffness maps are degraded, making motion artifacts difficult to recognize visually before generating the stiffness maps.

The evaluation of subject motion impact on mechanical properties has context-dependent usability. In studies targeting subtle biological effects, such as a study that has shown that whole brain stiffness changes 0.3% per year in younger and middle-aged groups and 0.4% per year in older adults [31], or a study linking hippocampal subfield stiffness to memory performance [32], a motion-induced stiffness error of even 1%–5% may render a dataset unusable. Previous MRE work has found significant cerebrum stiffness differences of approximately 11% in people with Alzheimer’s disease [33], of 20.46% in affected regions in people with multiple sclerosis [34], and changes of 6.0% in gray matter stiffness in children with cerebral palsy [21]. Given the subtle mechanical differences in some of these clinical populations, even small errors from subject motion can impact conclusions drawn. In contrast, for certain clinical applications where lesions exhibit large mechanical contrast, stiffness maps may remain diagnostically useful despite moderate motion error, particularly when motion-correction methods can reduce that error. Therefore, it is important that researchers assess and report the degree of subject motion in their datasets prior to publication. While motion correction improves image quality and reduces stiffness error, residual errors may remain in cases with severe motion. In such cases, the corrected data may still not meet the requirements for studies targeting subtle biological effects. Nevertheless, motion correction can extend the range of usable scans by recovering data that would otherwise be discarded, particularly in cases of small to intermediate motion.

Experiment 1 demonstrates that errors arising from subject motion in brain MRE depend not only on motion amplitude, but also on the temporal structure of motion. Erratic motion caused the greatest instability in displacement fields and stiffness maps, whereas move-once-return motion produced the smallest errors by maintaining overall temporal consistency. Drift motion and move-once-stay led to intermediate levels of degradation. These results demonstrate that sudden, repeated motions are more harmful than a single large movement. Further, inaccuracies are not determined by subject motion amplitude alone, but by the interaction between motion pattern and encoding strategy. The 8-offset dataset was more tolerant of transient motion, whereas the 4-offset dataset was more robust to sustained motion, likely reflecting differences in temporal sampling and accumulation of offset-specific phase inconsistencies.

The proposed approach is a flexible framework that can be integrated with existing reconstructions. Here we incorporate it into a 3D multiband, multishot MRE sequence with field-inhomogeneity correction [20], phase-error correction [22], and parallel imaging [23]. By embedding rigid-body transformations directly into the forward and adjoint operators, the pipeline preserves operator consistency and enables motion correction to be performed within the same iterative framework that already supports field-map compensation, phase-error modeling, parallel imaging, and non-Cartesian sampling, thereby treating motion as part of the physical signal model rather than an after-the-fact adjustment. This integration is essential for preserving quantitative fidelity, as modern high-efficiency acquisitions, including multiband spiral [35], 3D EPI [36], compressed sensing [37], spatiotemporal encoding [18], and advanced inversion frameworks [38, 39], are increasingly sensitive to even small misalignments in the complex domain. As advanced sampling strategies such as variable-density spirals [40], wave-CAIPI [41], and model-based reconstructions [42] continue to push MRE toward higher resolution and greater efficiency, motion-aware reconstruction will become foundational for reliable stiffness estimation.

The proposed strategy utilizes navigator-based motion estimation and reconstruction-based motion compensation. Other volumetric navigator-guided approaches have demonstrated similar performance on brain MRI. A 3D fat navigator-guided retrospective correction study has shown overall improvement across minor to severe motion artifacts in brain MRI of patients with tumors [9], demonstrating the clinical efficacy of navigator-based motion correction. Other than explicit navigator acquisition, a self-navigated framework that estimates in-plane shifts between adjacent echo trains via subsampled phase correlation in k-space and retrospectively applies linear phase ramps for correction has been validated on brain MRI [8], showing sharpened anatomical boundaries and improved image quality. A similar self-navigation strategy was employed in a variable-density spiral sequence, where an oversampled k-space center can be used as an inherent 2D navigator to reconstruct a low-resolution phase map for each spiral interleaf, which is then iteratively subtracted in image space to correct motion-induced phase errors between shots, thereby correcting shifts even with high-resolution brain images [24]. Other navigator-free motion-correction approaches that rely on the implicit image properties to enable motion-resolved images have been proposed. For example, a multitasking framework that models head motion as an additional time dimension within a low-rank tensor reconstruction has been designed, achieving retrospective motion-robust quantitative mapping without explicit registration-based correction, and is able to restore motion-free images close to reference images [10]. Cordero-Grande et al. proposed a 3D rigid-body motion-corrected reconstruction for multi-shot multi-slice brain MRI that jointly estimates per-shot 3D motion parameters and the underlying image within a unified forward model and incorporates slice-profile-based through-plane motion correction, which demonstrates diminished motion artifacts in neonatal brain imaging [15]. However, these methods have largely been developed for magnitude-based anatomical imaging, and their impact on phase-sensitive MRE, where residual motion errors directly bias viscoelastic property estimation, has not been established.

While this paper focuses on brain MRE, the underlying principles of rigid-body motion-induced phase inconsistency and its impact on stiffness estimation are likely applicable to MRE in other regions of the body. However, the nature of motion differs across anatomical regions. In body MRE, motion is often dominated by respiration [43], cardiac pulsation [44], or organ deformation, which may introduce additional challenges, particularly for non-rigid motion that is not captured by the current framework. Therefore, while the general impact of motion and the benefits of motion-aware reconstruction are expected to extend to other MRE applications, further work is needed to evaluate and adapt these methods for more complex situations.

Despite its strengths, the proposed method has several limitations. Navigator-based motion estimation may be less reliable in regions with extremely low SNR [9] or in cases of non-rigid or through-plane motion that cannot be captured by 2D rigid-body registration. In addition, sub-voxel-scale motion remains difficult to estimate and correct, as navigator image registration cannot capture motion at the sub-voxel level. Furthermore, although the method has exhibited great potential in motion correction, its post-acquisition nature requires additional steps to estimate and correct motion during or after image reconstruction, which is not optimal in clinical settings. Future work should consider conducting *k*-space motion correction to allow sub-voxel compensation and prospective motion correction [12] to advance toward real-time solutions for clinical use. Finally, the clinical dataset presented here used simulated rigid-body motion and should be interpreted as a controlled benchmark rather than a full model of in vivo motion, since real head motion may also alter driver coupling and the underlying wave field.

## 6 | Conclusion

The proposed method provides a robust and flexible motion correction framework for brain MRE across both single-shot and multi-shot acquisitions. We show that stiffness accuracy can deteriorate even when magnitude images appear normal, underscoring that motion must be identified and corrected to preserve the quantitative integrity of MRE. By estimating motion from magnitude images and compensating in the complex domain, motion correction enhances anatomical image quality while increasing the reliability of mechanical measures, thereby improving data quality in both research and clinical MRE. This method underscores the need for dedicated motion-detection and correction strategies as MRE acquisition methods become more advanced and supports broader clinical adoption of brain MRE.

## Supplementary Material

Supplemental Information

Additional supporting information can be found online in the Supporting Information section. Table S1: Percent improvement in stiffness map quality in gray matter and white matter regions following motion correction for multi-shot (*n* = 9) and single-shot (*n* = 9) MRE acquisitions. Improvements were calculated relative to the uncorrected reconstruction for each scan. Figure S1: COMET pipeline with all intermediate steps. (a) Single-shot post-reconstruction correction and reorientation. (b) Multi-shot iterative motion-aware reconstruction and reorientation. Figure S2: Subject motion obscures the visibility of tumor stiffness without motion correction. T2-weighted MRE magnitude images, displacement maps, and stiffness maps are shown for no-motion, simulated-motion at 10mm of translation, and motion-corrected conditions. Motion blurred the tumor boundaries and corrupted the stiffness map, while motion correction restored sharpness and recovered stiffness. Figure S3: Comparison of motion-corrected multishot spiral stiffness maps with and without displacement-vector reorientation. Representative magnitude images and stiffness maps are shown for datasets processed using image alignment alone or image alignment followed by displacement-vector reorientation. The corresponding average stiffness error (AVSE) for each dataset is reported relative to the no-motion reference. Figure S4: Comparison of motion-corrected single-shot EPI stiffness maps with and without displacement-vector reorientation. Representative magnitude images and stiffness maps are shown for datasets processed using image alignment alone or image alignment followed by displacement-vector reorientation. The corresponding average stiffness error (AVSE) for each dataset is reported relative to the no-motion reference.

## Figures and Tables

**FIGURE 1 |:**
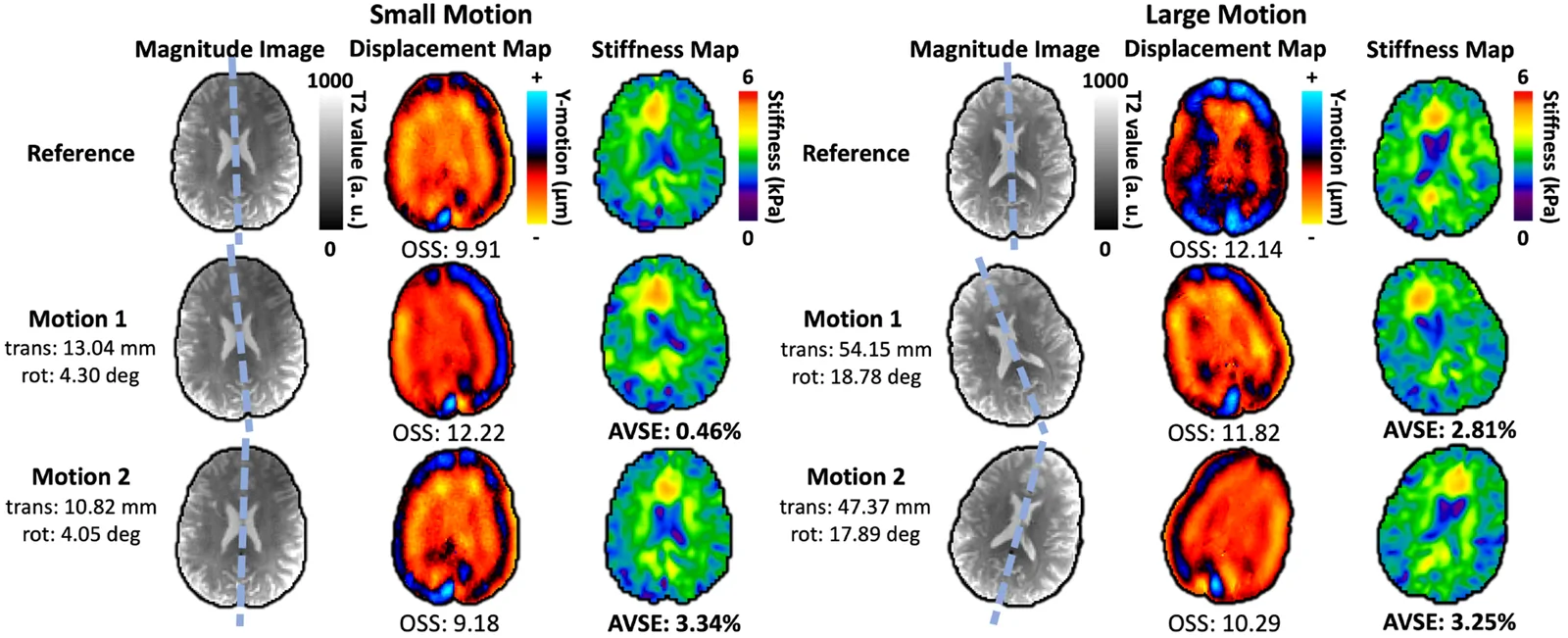
Wave pattern variability. Changes in head position relative to the passive driver alter measured stiffness distributions, ranging from 0.46% to 3.34% across small (10.82–13.04 mm translation and 4.05°–4.30° rotation) and large reposition conditions (47.37–54.15 mm translation and 17.89°–18.78° rotation).

**FIGURE 2 |:**
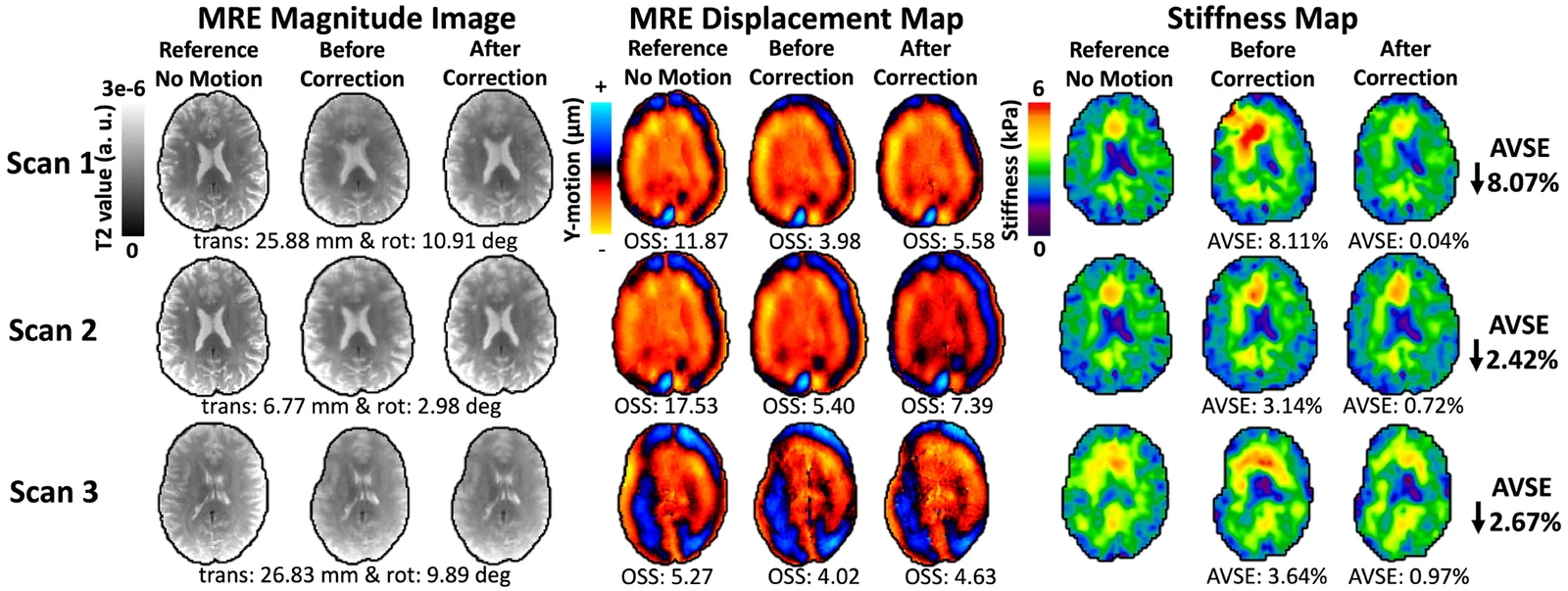
Representative examples in which motion correction restored stiffness accuracy in multishot spiral MRE to within 1.5% AVSE. T_2_-weighted MRE magnitude images, displacement maps, and stiffness maps are shown for three subjects under no-motion, with-motion, and motion-corrected conditions. The corrected stiffness maps maintained close agreement with the no-motion reference, with absolute voxelwise stiffness error (AVSE) remaining below 1.5% across all scans.

**FIGURE 3 |:**
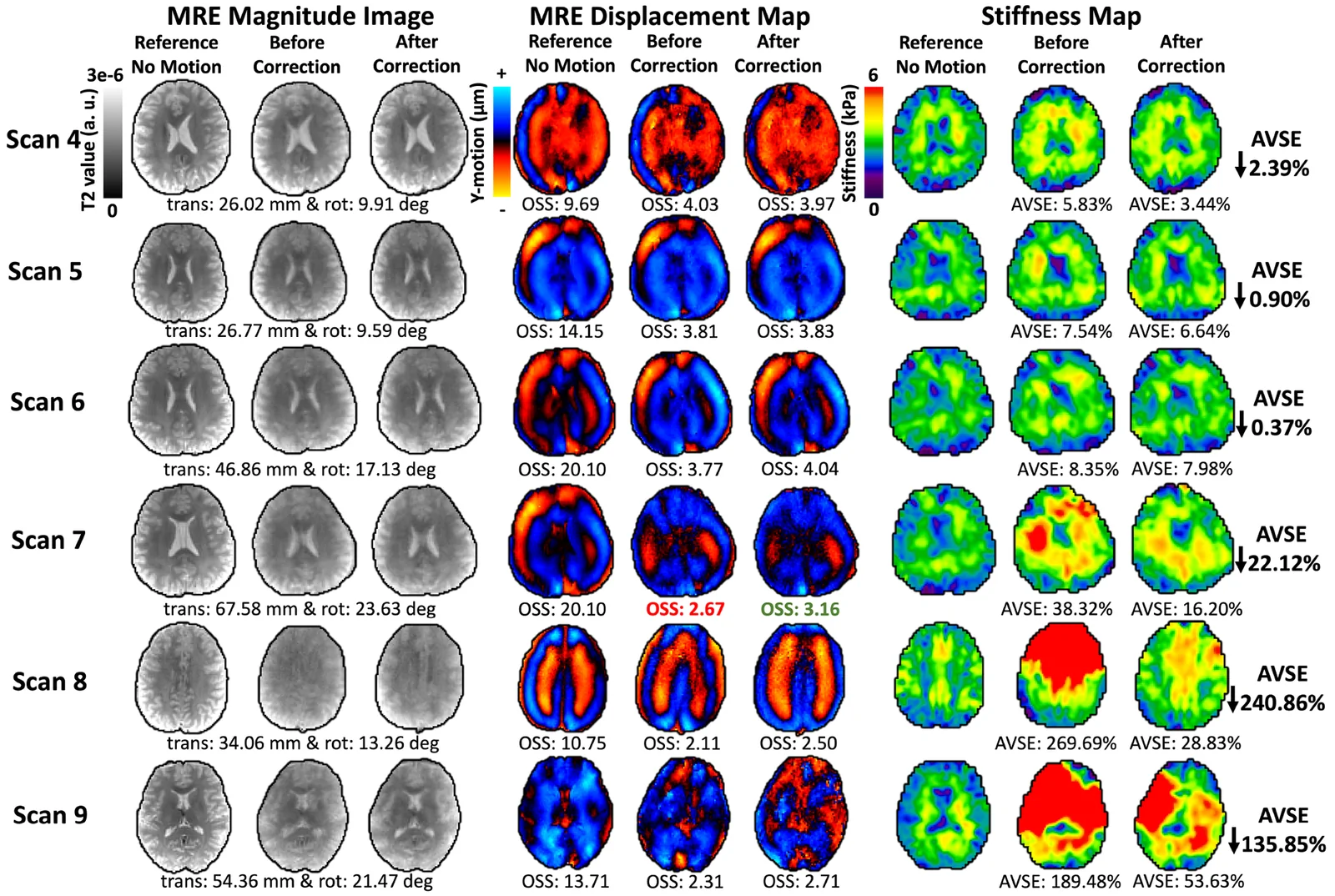
Stiffness maps are improved in all tested multishot spiral MRE. T_2_-weighted MRE magnitude images, displacement maps, and stiffness maps are shown for six subjects under no-motion, with-motion, and motion-corrected conditions. Motion correction consistently improves image alignment, displacement-map coherence, and stiffness-map agreement with the no-motion reference, although recovery was incomplete in scans with the greatest motion. The scan with the largest initial motion (Scan 7) and low pre-correction OSS-SNR was restored to OSS-SNR > 3 after correction, highlighted in red and green.

**FIGURE 4 |:**
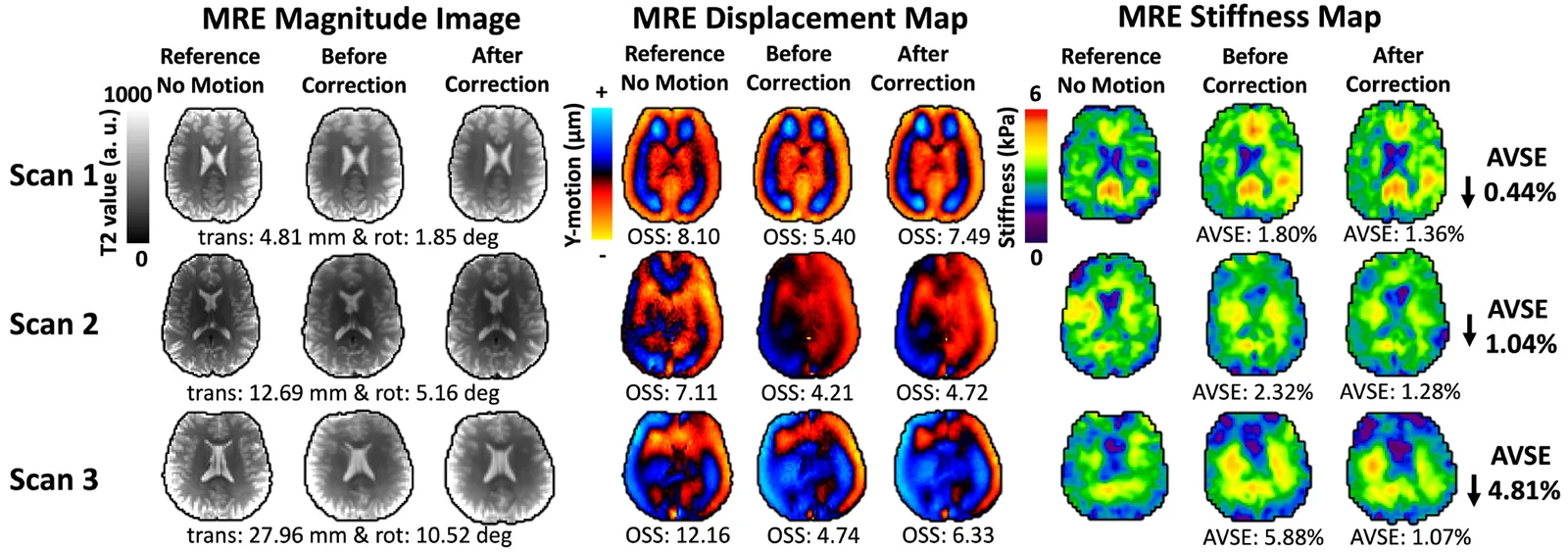
Representative examples in which motion correction restored stiffness accuracy in single-shot EPI MRE to within 1.5% AVSE. T_2_-weighted MRE magnitude images, displacement maps, and stiffness maps are shown for three subjects under no-motion, with-motion, and motion-corrected conditions. The corrected stiffness maps showed close agreement with the no-motion reference, with absolute voxelwise stiffness error (AVSE) remaining below 1.5% across all scans.

**FIGURE 5 |:**
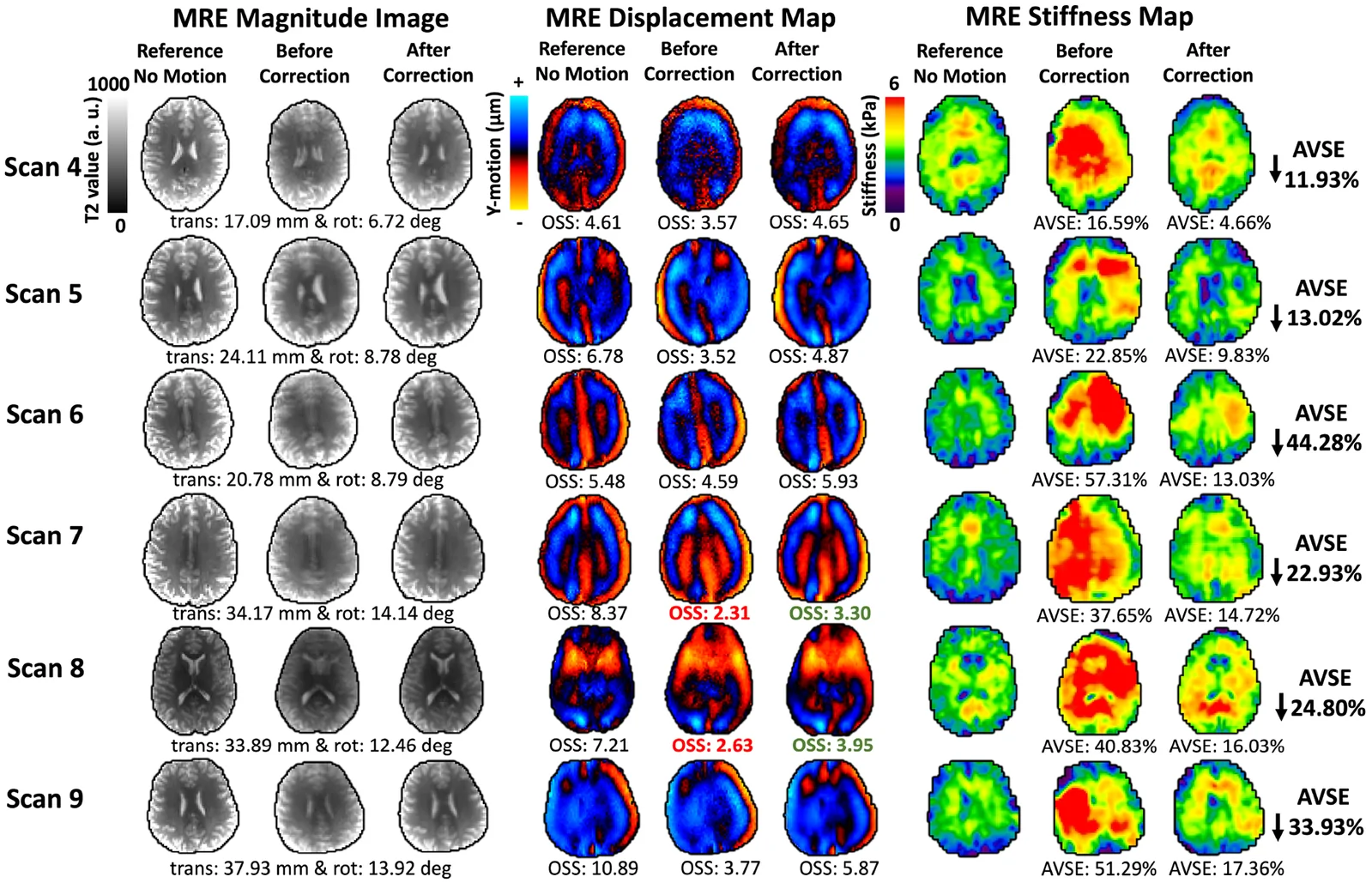
Stiffness maps are improved in all tested single-shot EPI images. T_2_-weighted MRE magnitude images, displacement maps, and stiffness maps are shown for six subjects under no-motion, with-motion, and motion-corrected conditions. Correction consistently improved image alignment, displacement-map coherence, and stiffness-map agreement with the no-motion reference, although recovery was incomplete in scans with the greatest motion. All scans with OSS-SNR < 3 were recovered to OSS-SNR > 3 after correction, highlighted in red and green.

**TABLE 1 |:** Subject motion patterns result in varying levels of stiffness map error.

A) 8-offset Image Motion Simulation												
NRMSE (%)	Drift Motion			Erratic Motion			Move Once and Return			Move Once and Stay		

Directions	x	y	rot	x	y	rot	x	y	rot	x	y	rot

**1mm / 2 deg**	2.51%	2.83%	3.96%	2.56%	2.62%	3.93%	0.49%	0.50%	0.53%	2.65%	3.32%	5.25%
**2.5mm / 5 deg**	6.17%	6.27%	8.94%	6.47%	6.41%	8.54%	0.89%	0.94%	1.05%	8.11%	7.01%	9.14%
**5.0mm / 10 deg**	13.39%	10.36%	21.17%	15.61%	13.96%	18.56%	1.94%	1.81%	1.87%	11.55%	16.23%	30.81%
**7.5mm / 15 deg**	14.72%	15.06%	20.24%	44.61%	31.69%	69.00%	2.85%	2.86%	2.55%	16.50%	27.29%	22.65%
**10.0mm / 20deg**	16.83%	20.70%	27.20%	198.06%	82.86% :	343.59%	2.95%	4.40%	3.37%	19.53%	45.69%	36.41%

AVSE (%)	Drift Motion			Erratic Motion			Move Once and Return			Move Once and Stay		

Directions	x	y	rot	x	y	rot	x	y	rot	x	y	rot

**1mm / 2 deg**	0.84%	0.81%	1.05%	0.98%	0.59%	1.16%	0.03%	0.08%	0.04%	0.91%	0.86%	1.70%
**2.5mm / 5 deg**	1.54%	1.15%	3.06%	2.60%	1.28%	3.25%	0.07%	0.21%	0.25%	2.10%	1.37%	3.85%
**5.0mm / 10 deg**	3.58%	2.53%	8.02%	7.56%	4.86%	8.92%	0.43%	0.66%	0.49%	4.02%	6.25%	11.87%
**7.5mm / 15 deg**	4.47%	4.61%	10.28%	19.72%	12.48%	23.33%	0.63%	0.91%	0.41%	6.47%	12.27%	12.16%
**10.0mm / 20deg**	5.68%	8.66%	14.94%	47.14%	27.87%	55.30%	0.52%	1.71%	0.40%	7.21%	19.27%	17.44%

B) 4-offset Image Motion Simulation												
NRMSE (%)	Drift Motion			Erratic Motion			Move Once and Return			Move Once and Stay		

Directions	x	y	rot	x	y	rot	x	y	rot	x	y	rot

**1mm / 2 deg**	2.43%	2.94%	3.95%	1.99%	2.03%	3.24%	0.78%	0.91%	1.02%	2.50%	3.21%	4.12%
**2.5mm / 5 deg**	5.36%	6.58%	8.20%	4.23%	4.31%	7.25%	1.81%	1.74%	2.16%	5.57%	6.98%	8.93%
**5.0mm / 10 deg**	9.36%	11.00%	13.65%	9.32%	10.25%	12.09%	3.32%	3.32%	3.87%	10.87%	13.61%	15.28%
**7.5mm / 15 deg**	13.33%	15.72%	18.62%	17.74%	16.81%	20.41%	5.06%	6.52%	4.93%	15.84%	22.53%	20.50%
**10.0mm / 20deg**	16.78%	20.06%	24.42%	30.64%	23.56%	98.61%	6.49%	8.24%	6.73%	19.72%	40.52%	48.60%

AVSE (%)	Drift Motion			Erratic Motion			Move Once and Return			Move Once and Stay		

Directions	x	y	rot	x	y	rot	x	y	rot	x	y	rot

**1mm / 2 deg**	0.68%	0.70%	0.91%	0.62%	0.53%	1.05%	0.02%	0.10%	0.12%	0.65%	0.66%	1.07%
**2.5mm / 5 deg**	1.06%	0.90%	2.11%	1.33%	1.49%	2.71%	0.20%	0.27%	0.52%	1.10%	1.02%	3.12%
**5.0mm / 10 deg**	2.25%	2.28%	4.96%	3.40%	4.10%	4.97%	0.74%	1.02%	1.21%	3.02%	4.44%	7.05%
**7.5mm / 15 deg**	3.86%	4.14%	8.62%	8.22%	5.87%	9.31%	1.09%	1.48%	1.47%	5.39%	9.27%	10.54%
**10.0mm / 20deg**	5.63%	7.11%	12.69%	14.44%	8.64%	27.93%	1.72%	2.20%	1.84%	6.95%	14.71%	17.79%

*Note:* Retrospective in-plane translations (1–10mm) and rotations (2° – 20°) were applied to raw EPI data under four motion patterns (drift, erratic, move-and-return, and move-and-stay). Resulting stiffness errors were evaluated for (A) an 8-offset scan and (B) a 4-offset subset. Erratic motion produced the largest error, while move-and-return produced the least. The 8-offset was more robust to transient motion, while the 4-offset was more robust to sustained motion due to fewer acquisitions.

## Data Availability

The motion correction pipeline is available at https://github.com/zhuoyu73/COMET.
