# Supplementary material for: Iterative Navigator-Based Retrospective Motion Correction for Multi-Shot Brain MR Elastography and Evaluation of Single-Shot Post-Reconstruction Correction
Source: Magn Reson Med. Author manuscript; Available in PMC 2026 Nov 9. (PMC13747630; doi:10.1002/mrm.70625)
Supplement: Supplementary file 1 [file NIHMS2218149-supplement-Supplemental_Information.docx]

**Iterative Navigator-Based Retrospective Motion Correction for Multi-Shot Brain MR Elastography and Evaluation of Single-Shot Post-Reconstruction Correction**

Zhuoyu Shi^1^, Alex M Cerjanic^2^, Grace McIlvain^1,3*^

1. Department of Biomedical Engineering, Fu Foundation School of Engineering and Applied Science, Columbia University, New York, New York, USA

2. Department of Neurology, Massachusetts General Hospital, Boston, MA, United States

3. Department of Radiology, Vagelos College of Physicians and Surgeons, Columbia University, New York, New York, USA

* Corresponding author.

**Supplemental Information**

| **Multi-Shot** | **Gray Matter Improvement** | **White Matter Improvement** | **Single-Shot** | **Gray Matter Improvement** | **White Matter Improvement** |
| --- | --- | --- | --- | --- | --- |
| **Scan 1** | 9.94% | -1.07% | **Scan 1** | 5.43% | 7.96% |
| **Scan 2** | 3.36% | 1.09% | **Scan 2** | 1.15% | 2.88% |
| **Scan 3** | 5.76% | -5.69% | **Scan 3** | 2.80% | -9.25% |
| **Scan 4** | 3.45% | -0.26% | **Scan 4** | 6.64% | 21.51% |
| **Scan 5** | 2.10% | 0.43% | **Scan 5** | 5.49% | 12.48% |
| **Scan 6** | 1.68% | 1.93% | **Scan 6** | 41.23% | 57.68% |
| **Scan 7** | 5.19% | 14.57% | **Scan 7** | 21.27% | 24.97% |
| **Scan 8** | 333.93% | 99.85% | **Scan 8** | 22.36% | 39.83% |
| **Scan 9** | 143.28% | 160.75% | **Scan 9** | 29.39% | 49.97% |

***Table S1: Percent improvement in*** ***stiffness map quality in gray matter and white matter*** ***regions following motion correction for multi-shot (n = 9) and single-shot (n = 9) MRE acquisitions.*** *Improvements were calculated relative to the uncorrected reconstruction for each scan.*

***Figure S1: COMET pipeline with all intermediate steps.*** *a) Single-shot post-reconstruction correction and reorientation. b) Multi-shot iterative motion-aware reconstruction and reorientation.*

***Figure S2: Subject motion obscures the visibility of tumor stiffness without motion correction.*** *T2-weighted MRE magnitude images, displacement maps, and stiffness maps are shown for no-motion, simulated-motion at 10mm of translation, and motion-corrected conditions. Motion blurred the tumor boundaries and corrupted the stiffness map, while motion correction restored sharpness and recovered stiffness.*

***Figure S3: Comparison of motion-corrected multishot spiral stiffness maps with and without displacement-vector reorientation.*** *Representative magnitude images and stiffness maps are shown for datasets processed using image alignment alone or image alignment followed by displacement-vector reorientation. The corresponding average stiffness error (AVSE) for each dataset is reported relative to the no-motion reference.*

***Figure S4: Comparison of motion-corrected single-shot EPI stiffness maps with and without displacement-vector reorientation.*** *Representative magnitude images and stiffness maps are shown for datasets processed using image alignment alone or image alignment followed by displacement-vector reorientation. The corresponding average stiffness error (AVSE) for each dataset is reported relative to the no-motion reference.*
